# Young Children’s Development of Fairness Preference

**DOI:** 10.3389/fpsyg.2016.01274

**Published:** 2016-08-30

**Authors:** Jing Li, Wen Wang, Jing Yu, Liqi Zhu

**Affiliations:** ^1^Key Laboratory of Behavioral Science, Institute of Psychology, Chinese Academy of SciencesBeijing, China; ^2^Human Development and Family Studies, College of Social Science, Michigan State University, East LansingMI, USA; ^3^Department of Psychology, University of Maryland, Baltimore County, BaltimoreMD, USA

**Keywords:** fairness, inequity aversion, young children, forced choice paradigm, distribution

## Abstract

Fairness is one of the most important foundations of morality and may have played a key role in the evolution of cooperation in humans beings. As an important type of fairness concern, inequity aversion is the preference for fairness and the resistance to inequitable outcomes. To examine the early development of fairness preference in young children, sixty 2- and 3-year-old children were recruited to examine young children’s preferences for fairness using a forced choice paradigm. We tested how toddlers acted when they took charge of distributing resources (two candies) to themselves and others and when they were the recipients of both other-advantageous distribution and self-advantageous distribution. Different alternative options were paired with the same fair option in the two conditions. In the other-advantageous condition, children had fewer resources in the alternative options than others, whereas their resources in the alternative options were greater than others’ in the self-advantageous condition. The results showed that more children displayed fairness preferences when they distributed resources between two friends than when they distributed resources between a friend and themselves. In both scenarios, 3-year-old children were more likely to demonstrate fairness preference than 2-year-old children. The findings suggest that inequity aversion develops in young children and increases with age over the course of early childhood. When they were recipients, there was a trend in young children’s preference for fairness in the other-advantageous condition compared with the self-advantageous condition. This suggests that children might tend to be more likely to display inequity aversion when they are in a disadvantageous position.

## Introduction

As the philosopher John Rawls noted, ‘the fundamental idea in the concept of justice is fairness’ (Rawls, 1958). Fairness is one of the most important foundations of morality in both older (Piaget, 1965; Kohlberg, 1969) and newer (Haidt and Graham, 2007) theories of moral psychology. Unsurprisingly, fairness concerns have received much attention in the areas of behavioral economics (Fehr and Schmidt, 1999), psychology (Declerck et al., 2009) and evolutionary biology (Fehr and Fischbacher, 2003; Bräuer et al., 2006). Human beings have a substantial desire for fairness and show strong aversions to inequity (Fehr and Schmidt, 1999). Even third parties who do not personally suffer from the inequity will punish others for unfair behavior to achieve fairness (Fehr and Fischbacher, 2004; Dawes et al., 2007; Fehr et al., 2008b). Inequity aversion and the rejection of unfairness are considered essential for maintaining cooperation and reducing opportunities for free riders (Kogut, 2012) and thus may have played a key role in the evolution of cooperation in humans (Fehr and Schmidt, 1999; Fehr and Fischbacher, 2003).

A wealth of empirical evidence gathered by experimental economists and psychologists suggests that a high percentage of people are strongly motivated by other-regarding preferences and that concerns for fairness and reciprocity cannot be ignored in social interactions (Fehr and Schmidt, 2003). Theories such as the dual concern model (Pruitt and Rubin, 1986) and the social utility model (Loewenstein et al., 1989) suggest that people prefer to consider the other’s benefits in their distribution decisions in addition to the wish to maximize one’s own utility. According to the social utility model, people feel more comfortable and experience greater satisfaction with the equal distribution of resources than with inequitable allocations, even when those inequities are self-advantageous (Loewenstein et al., 1989; Kogut, 2012). In addition, the ERC (Equity, Reciprocity, and Competition) model, proposed by Bolton and Ockenfels (2000), highlights the concern for one’s relative position (competition) in social interactions.

As an important type of fairness concern, inequity aversion is one’s preferences against receiving either more or less than someone else (Fehr and Schmidt, 1999). Although people are especially motivated to achieve fairness when the inequity is to one’s own disadvantage, there is some evidence showing that the desire for fairness is strong even when the inequity is to one’s advantage (Haynes and Gilovich, 2010). As proposed by Fehr and Schmidt (1999), there are two kinds of inequity aversion (IA): one is disadvantageous IA, in which another individual receives more than oneself, and the other is advantageous IA, in which one receives more than another individual (Hatfield et al., 1978). It is argued that adults will sacrifice their own benefits to eliminate inequalities they view as unfair by punishing unequal outcomes, both when they are offered more resources than a social partner (advantageous IA) and when they are offered relatively fewer resources (disadvantageous IA) (Fehr and Schmidt, 1999; Camerer, 2003; Fehr and Fischbacher, 2003; Dawes et al., 2007). Therefore, the sense of fairness has at least two distinct components, including a desire to be fair and a desire to signal to others that they are fair (Shaw et al., 2014).

The research on sharing behavior in adults suggests that people tend to share their resources and feel better with an equal distribution even when no strategic considerations exist (Loewenstein et al., 1989). Adult preferences for equity using strategic and economic games have been investigated in different cultures (Fehr and Fischbacher, 2003; Henrich et al., 2005; Camerer and Fehr, 2006), and substantial behavioral variability across social groups was found; theories of both cultural evolution as well as gene–culture co-evolution are assumed to explain the interaction between altruists and selfish individuals and individual heterogeneity in altruism. However, less is known about how the preference for fairness develops in childhood.

Some researchers have found that children begin to understand fairness between the ages of 4 and 6 years old. Lane and Coon (1972) found that 4-year-olds generally distributed stickers selfishly to a fictitious partner, whereas most 5-year-olds distributed stickers more equally to fictitious partner. Similarly, Damon (1975) found that 4-year-old children often confused fairness with their own desires in hypothetical dilemmas, while 5-year-olds began to focus on strict equality. Several studies using behavioral economics methods (e.g., dictator game and ultimatum game) (Gummerum et al., 2008) have found that 3- to 4-year-old children would like to share some resources, but a preference for equal distribution does not emerge until 7 years of age or later (Harbaugh et al., 2003; Benenson et al., 2007). Fehr et al. (2008a) found that most 3- to 4-year-old children behaved selfishly, whereas most 7- to 8-year-olds preferred equitable resource distribution, suggesting that children under 6 years old behaved primarily based on selfish desires than on fairness concerns. Children’s preferences for equal distributions increased with age (Benenson et al., 2007; Blake and Rand, 2010; Gummerum et al., 2010). Children between 6 and 8 years old begin to incur costs to avoid inequality such as discarding a resource to avoid an unequal resource distribution (Blake and McAuliffe, 2011; Shaw and Olson, 2012). Similarly, Hook and Cook (1979) suggested that 8-year-old children are more willing to bear costs to achieve fairness than 3-year-old children. In sum, fairness preferences develop late in children, at the minimum, 6 or 7 years of age is when children distribute resources equally; however, they develop an increasing preference for fairness throughout the course of childhood (Fehr et al., 2008a; Shaw et al., 2014).

On the other hand, recent research on infants and preschoolers challenges this notion, showing that knowledge of fairness and fair behavior emerges earlier than expected. Infants who are 16 months old are able to pay attention to equality in resource distribution by expecting resources to be allocated equally among recipients using an index of looking time and manual choices provided (Geraci and Surian, 2011; Schmidt and Sommerville, 2011). Infants at 19 months of age looked longer when one puppet received both items than when each puppet received one item using a looking-time paradigm, suggesting that 19-month-old infants expected an equal allocation (Sloane et al., 2012). Similar experiments have shown that even 15-month-olds expect equal resource allocations (Sommerville et al., 2013). In addition, a substantial amount of empirical evidence suggests that 3- to 4-year-old children already show a preference for fairness in different contexts. Three-year-old children might notice and be averse to disadvantageous inequality in distributions (Birch and Billman, 1986). Children aged 3.5–4 years old show a strong preference for giving one object equally to each doll (Olson and Spelke, 2008). Children aged 4 years old favor equality over giving others more resources and prefer fairness over generosity in some circumstances (Kenward and Dahl, 2011). Around the same age, they exhibit negative emotional reactions to unequal distribution and are willing to incur costs to ensure that they do not have less than others (LoBue et al., 2011). Phenomena on inequity aversion in young children have been reported from the developmental perspective, suggesting an early onset of inequity aversion (Blake and McAuliffe, 2011; Paulus et al., 2013).

The existing empirical evidence on whether 3- to 4-year-old and younger children prefer fairness (i.e., experience inequity aversion) is not conclusive, indicating the need for more studies on the ontogenetic origins of fairness. Specifically, older studies (e.g., Damon, 1975) suggested that children do not develop fairness preferences until they are 5 years old or older, whereas recent studies on infants and preschoolers showed that a fairness preference emerges even in children younger than 2 years old. The discrepancy might be due to the differences in both the paradigms and contexts. For example, infant studies have adopted a preferential looking-time paradigm, which is effective in gaining insight into the young mind by assessing the characteristics of infants’ innate cognitive faculties (Cohen and Cashon, 2003), whereas older studies (e.g., Lane and Coon, 1972 and Damon, 1975) used fictitious partners or hypothetical dilemmas, which differ from real distribution contexts. In addition, the inconsistent results on the preference for fairness in young children might be due to the fact that some research focused on the knowledge of fairness, whereas other research focused on fair behavior. It is argued that having knowledge about the principles of fairness does not guarantee that one will use them when making decisions (Blake et al., 2014). For example, when given a set of stickers, children between 3 and 8 years of age recognize that sharing half of the stickers with an absent child would be the right thing to do, but only 7- to 8-year-old children actually distribute the stickers equally (Smith et al., 2013). This gap between knowledge of fairness and fair behavior might occur because younger children cannot inhibit their desires for resources and thus fail to follow the fairness norms they already know (Blake et al., 2014). The desire to maintain an advantageous position compared with one’s peers is also adopted to explain this gap (Blake et al., 2014).

It is argued that the development of children’s aversion to disadvantageous and advantageous inequity is asymmetrical (McAuliffe et al., 2013). Children as young as 3 years old accept allocations that would place themselves in a relatively advantageous position and reject those that would put them at a relatively disadvantageous position (exhibiting aversion to disadvantageous inequity) (Fehr et al., 2008a; Takagishi et al., 2010; Blake and McAuliffe, 2011; LoBue et al., 2011; Sheskin et al., 2014). However, children do not develop an aversion to advantageous inequity until 8 years old (Blake and McAuliffe, 2011; Shaw and Olson, 2012). In addition, experiments on non-human animals have demonstrated that domestic dogs (Range et al., 2009) and non-human primates (Jensen et al., 2006; Proctor et al., 2013) are both sensitive to disadvantageous inequity, but no evidence supports the notion that non-human animals perform aversion to advantageous inequity. These findings from human children and non-human animals might suggest that separate developmental mechanisms underlie these two forms of inequity aversion (McAuliffe et al., 2013).

Despite the demonstrated cross-cultural variability in young children’s (Rao and Stewart, 1999; Rochat et al., 2009) and adults’ (Henrich et al., 2005) resource distribution behaviors, a potentially universal inclination for inequity aversion is worth noting (Paulus, 2015). More fairness in distributive justice is evident in 3- to 5-year-old children growing up in small-scale urban and traditional societies that are thought to promote more collective values (Rochat et al., 2009). Rao and Stewart (1999) found that 4-year-old Chinese children showed more spontaneous sharing than Indian children, while Indian children performed substantially more passive sharing. Moreover, Zhu et al. (2008) found that Chinese children and adolescents of 9, 12, and 14 years of age displayed a decreasing preference for fairness with age in a dictator game as the proposer, while many studies in Western cultures found an increasing tendency in fairness preference with age (Eisenberg et al., 1998; Fehr et al., 2008a; Shaw et al., 2014). Cultures that have a scarcity of resources and a greater power distance (i.e., put less emphasis on equality) and those that are less individualistic (i.e., put less emphasis on the rights of individuals) (Hoftede et al., 2010) constitute the cornerstone of human inequity aversion (Paulus, 2015). Traditional Chinese culture especially emphasizes equity, for example, Confucius argued that “Do not worry about poverty, but rather about the unequal distribution of wealth” (Confucius, 1980).

Previous fairness research has mostly relied on explicit measures, such as interviews and questionnaires, to indicate preference for fairness, which requires verbal responses from children (Premack, 2007). However, verbal reports may underestimate what children actually know, and thus behavioral observations might be a more effective way to examine fairness preferences in younger children (LoBue et al., 2011). Economists have argued that two simple fairness-related constructs, disadvantageous IA and advantageous IA, can be measured without verbal reports (Loewenstein et al., 1989; Fehr and Schmidt, 1999). A forced choice paradigm is suitable for children younger than 2 years old who have limited cognitive and linguistic competence (Hamlin et al., 2007) and is usually adopted to assess children’s responses to specific forms of inequity (Blake and McAuliffe, 2011). Specifically, children must choose between two options, for example, an advantageous allocation (two candies for me and none for you) and an equal allocation (e.g., one candy for me and one for you) that are presented simultaneously.

In sum, a clear inconsistency in whether young children display a fairness preference was found in previous studies, and little research on young children’s preference for fairness has been conducted in non-Western cultures. The goal of this study was to examine young Chinese children’s fairness preferences using an economic game paradigm. It is argued that the knowledge-behavior gap might lead to inconsistent results regarding when children are able to display a fairness preference, and children younger than 3 years might have knowledge of fairness but not display fair behavior. In the present study, we mainly focused on young children’s actual fair behavior (as the distributor). Scenarios about distributing resources as a third party and distributing resources between the self and another child were both included in our study. In addition to children’s fair behavior when they are in power as the distributors, children’s reactions when they are powerless recipients in dictator games (Eckel and Grossman, 1996) may provide a complementary picture of children’s preferences for fairness. A forced choice paradigm, which is sensitive to young children, was adopted in this study. In addition, cross-cultural variability in young children’s preference for fairness has been shown in previous studies. The preference for fairness in children 2–3 years of age in Chinese culture, a typical Eastern culture that emphasizes power distances, interdependence and group harmony, was examined in our study.

This study aimed to examine the development of fairness preference in 2- and 3-year-old Chinese children when they acted as distributors and as recipients. We hypothesized that young children would allocate resources equally when they were distributors, that the preference for fairness would increase with age, and that when they were recipients, children as young as 2 years old would prefer proposers who equally allocated resources.

## Experiment 1. Children’s Fairness Preference when they were Distributors

### Method

#### Participants

Sixty 2- and 3-year-old children were recruited from two child care centers in Baoding, Hebei province, China. One 2-year-old child was excluded from the analysis because he did not pay attention to the experimental procedures. The demographic characteristics of the children included in the analysis are shown in **Table 1**.

**Table 1:** Age and gender distribution across conditions with children as distributors.

Group	Distribute between self and friend		Distribute between two friends	
	Months	*n* (male)	Months	*n* (male)
2-year-old	28.1 ± 5.1	41 (23)	29.5 ± 4.5	26 (19)
3-year-old	40.5 ± 3.2	18 (10)	40.2 ± 4.0	11 (4)

The ethics committee of the Institute of Psychology, Chinese Academy of Sciences, approved our experiments. Informed consent forms were obtained from all children’s parents.

#### Procedure

A 2 (age: 2-year-old and 3-year-old) ^∗^ 2 (distribution conditions: distribute between self and friend and distribute between two friends) design was adopted in this experiment.

The experiments were conducted in a quiet room at the child care centers. In the warm-up phase, one human-like puppet and two similar rabbit puppets were introduced to the children. In the test phase, there were two conditions:

Condition 1: Distribute resources between self and friend. Children were offered two candies and were asked whether/how many they would like to give to another “friend,” which was a human-like puppet. The instructions were as follows. “Now I am giving you two candies. They are yours. Would you like to share with Lele (the human-like puppet)?” If the answer was “yes,” then the question “how many candies would you like to share with Lele?” was asked.

Condition 2: Distribute resources between two friends. Children were offered two candies and were asked to distribute both of these candies to two “friends,” which were two similar rabbit puppets. The puppets were set on two sides of a table. The distances between the two puppets and the children were the same. The instructions were as follows. “Here I have two candies, and they will be given to the two rabbits. I’d like to ask you to help me distribute the two candies to the rabbits. How many candies would you like to distribute to the rabbit on the left? How many candies to the rabbit on the right? Please place the candy in front of the rabbit.”

Children’s answers were coded as 0 (for an unfair distribution) and 1 (for a fair distribution).

### Results

**FIGURE 1:**
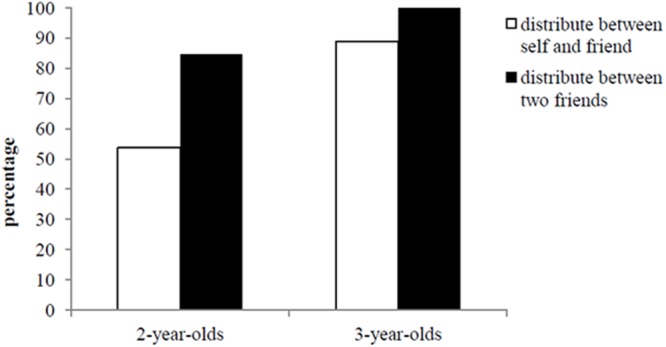
**Children’s fairness preference when they were the distributors**.

Children’s choices were first compared with the level of chance by binomial tests (see **Figure 1**). For 2-year-old children, their preference for fairness did not differ from chance when they distributed the resources between themselves and their friend (54% of 2-year-old children preferred fairness, *p* > 0.05). However, when they distributed the resources between two friends, they significantly preferred fairness compared to the level of chance (85% of 2-year-old children preferred fairness, *p* < 0.001). For 3-year-old children, they were more likely to prefer fairness above and beyond the level of chance both when they distributed the resources between themselves and their friend (89% of 3-year-old children preferred fairness, *p* < 0.001) and when they distributed the resources between two friends (100% of 3-year-old children preferred fairness). This suggested that 3-year-old children significantly preferred to distribute equally in both conditions, whereas 2-year-old children performed randomly when distributing resources between themselves and their friend but significantly preferred to distribute equally when distributing resources between two friends.

The binary logistic regression showed that more children displayed a fairness preference when they distributed resources between two friends than when they distributed resources between themselves and their friend [χ^2^(1) = 6.17, β = -1.56, *p* = 0.013]. Three-year-old children were more likely to prefer fairness than 2-year-old children regardless of whether they distributed resources between themselves and their friend or between two friends [*χ^2^*(1) = 5.66, β = -1.93, *p* = 0.017]. The interaction between distribution conditions and age was not significant [χ^2^(1) = 2.56, *p* = 0.109].

Experiment 1 revealed young children’s fairness preferences when they were the distributors. We were further interested in understanding young children’s fairness preferences when they were the recipients, which we examined in Experiment 2.

## Experiment 2. Children’s Fairness Preference when they were Recipients

### Method

#### Participants

The same 60 children participated in study 2 (completing four tasks in total). The order of the four tasks was counterbalanced. Three children were not included in the analysis because they did not finish the study, and one child was not included in the analysis because he did not pay attention to the procedures. The demographic characteristics of the children included in the analysis are shown in **Table 2**.

**Table 2:** Age and gender distribution across conditions with children as recipients.

Age group	(1,1)-(2,0)		(1,1)-(1,0)		(1,1)-(1,2)		(1,1)-(0,2)	
	Months	*n* (male)	Months	*n* (male)	Months	*n* (male)	Months	*n* (male)
2-year-old	28.2 ± 5.3	38 (23)	28.1 ± 5.2	40 (24)	28.1 ± 5.2	37 (21)	29.0 ± 4.8	24 (13)
3-year-old	40.7 ± 3.3	17 (9)	40.7 ± 3.4	16 (9)	40.7 ± 3.4	16 (9)	40.3 ± 3.5	14 (6)

*Four alternative options were paired with fair option (1,1) in these four conditions. For example, in the (1,1)-(2,0) scenario, the fair distributor allocated one gift to the subject, and one gift to his or her counterpart, whereas the unfair distributor gave the subject two gifts and nothing to the counterpart. Children were asked which distributor they preferred.*

The ethics committee at the Institute of Psychology, Chinese Academy of Sciences, approved our experiments. Informed consent forms from children’s parents were obtained for all subjects.

#### Procedure

A 2 (age: 2-year-old and 3-year-old) ^∗^ 4 (distribution conditions: (2,0)-(1,1), (1,0)-(1,1), (1,2)-(1,1), and (0,2)-(1,1)) design was adopted in this experiment.

The experiments were also conducted in a quiet room at the child care centers. The entire experiment was demonstrated as a puppet show to attract the young children’s attention. A forced choice paradigm was adopted to examine young children’s preference for fairness. In the warm-up phase, one human-like puppet was introduced to the children as their counterpart. The participating child and the human-like puppet were both the recipients. Four pairs of hand puppets were also prepared, and each hand puppet pair was the same except for their colors.

The experimenter used two hand puppets: one that distributed fairly between the child and the counterpart and another that distributed unfairly. The two hand puppets were placed in front of the child, and the distances between the child and these two hand puppets were the same. A control question of “how many gifts did the distributor give to you and your counterpart?” was first asked. Only children who answered the control question correctly moved into the test question phase; otherwise, the experimenter played the puppet show again. In the test question phase, the child was asked to choose which distributor he or she liked. Children’s answers were coded “1” for choosing the fair distributor and “0” for the unfair distributor.

There were four conditions that reflected different combinations of the fair versus unfair distributions (**Table 2**). In each condition, the fair puppet distributor gave both the participant and his or her counterpart one gift; the unfair puppet distributor gave the participant either more or less than his or her play partner. In the (1, 1)-(2, 0) scenario, the fair distributor allocated one gift to the subject and one gift to his or her counterpart; the unfair distributor gave the subject two gifts and nothing to the counterpart. The order of the equal and unequal distributions in each condition, the position of the fair distributor and the unfair distributor, and the sequence of the four conditions were all counterbalanced.

### Results

**FIGURE 2:**
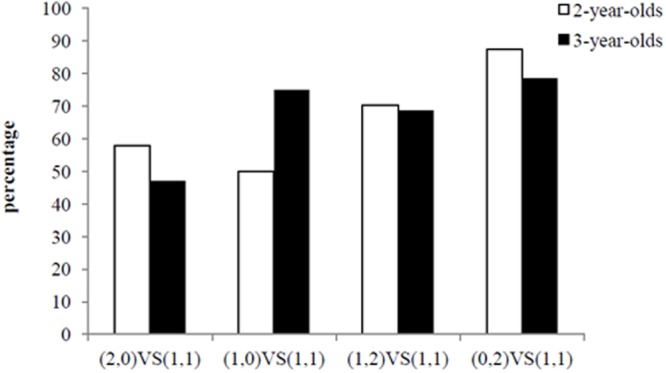
**The percentage of children preferring to fairness when others distributed resources for them and puppet**.

The children’s preferences for fairness are shown in **Figure 2**. Children’s choices were compared to the level of chance by binomial tests. For 2-year-old children, they significantly preferred fairness in the (1,2)-(1,1) condition [70% of 2-year-old children preferred fairness, *p* = 0.012] and the (0,2)-(1,1) condition [88% of 2-year-old children preferred fairness, *p* < 0.001], and their choices were similar to the level of chance in the (2,0)-(1,1) [58% of 2-year-old children preferred fairness, *p* > 0.05] and (1,0)-(1,1) [50% of 2-year-old children preferred fairness, *p* > 0.05] conditions. For 3-year-old children, they significantly displayed a fairness preference in the conditions of (1,0)-(1,1) [75% of 3-year-old children preferred fairness, *p* = 0.041] and of (0,2)-(1,1) [79% of 3-year-old children preferred fairness, *p* = 0.026], and their preference choices were similar to chance in the (2,0)-(1,1) [47% of 3-year-old children preferred fairness, *p* > 0.05] and (1,2)-(1,1) [69% of 3-year-old children preferred fairness, *p* > 0.05] conditions. This suggested that 2-year-old children significantly preferred fair choices when they were in the disadvantaged position in the alternative options but selected randomly when they were in the advantageous position in the alternative option. Three-year-old children significantly preferred fair choices when they were in the clearly disadvantaged position in the alternative option [(0,2)] and also preferred fairness when their own benefit in the fair option did not decrease compared with the alternative option [(1,0)-(1,1)], whereas they selected randomly when their own payoff in the fair option decreased compared with the alternative option [(2,0)-(1,1)].

Binary logistic regression showed that children’s preferences for fairness significantly differed across the distribution scenarios when they distributed sources with their friend [χ^2^(3) = 10.03, *p* = 0.018]. Preferences for fairness were comparable in the 2- and 3-year-olds when they distributed resources with their friend [χ^2^(1) = 0.04, β = -0.07, *p* = 0.837]. The interaction between the alternative options and age was not significant [χ^2^(3) = 3.86, *p* = 0.277]. The two age groups were then combined to further analyze the effect of the alternative options. Compared with the other three alternatives, the (0,2) option was more likely to motivate children to select a fair choice [(0,2) versus (2,0): χ^2^(1) = 8.15, β = -1.49, *p* = 0.004; (0, 2) versus (1,0): χ^2^(1) = 7.03, β = -1.38, *p* = 0.008; and (0,2) versus (1,2): χ^2^(1) = 2.40, β = -0.83, *p* = 0.121]. Young children showed similar fairness preferences between the conditions of (2, 0)-(1, 1) and (1, 0)-(1, 1) [χ^2^(1) = 0.08, β = 0.11, *p* = 0.780], as well as between the conditions of (2, 0)-(1, 1) and (1, 2)-(1, 1) [χ^2^(1) = 2.65, β = 0.66, *p* = 0.104]. This result demonstrated that the children were more likely to display a fairness preference when they were in a disadvantageous position, but not when they were in an advantageous position.

**FIGURE 3:**
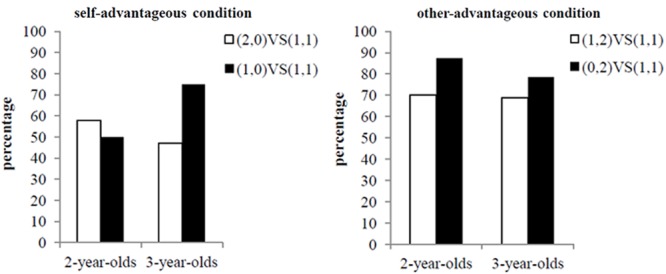
**Children’s fairness preference in conditions of self-advantageous and other-advantageous when they were the recipients**.

We combined the (2,0) and (1,0) conditions as self-advantageous conditions and (1,2) and (0,2) as other-advantageous conditions (showed in **Figure 3**). There was a trend in young children’s preference for fairness in the other-advantageous condition more so than in the self-advantageous condition [*F*(1,33) = 3.97, *p* = 0.055, $\eta_{p}^{2}$ = 0.11]. Moreover, in the self-advantageous condition, the interaction between the alternative options and age was significant [*F*(1,53) = 3.98, *p* = 0.05, $\eta_{p}^{2}$ = 0.07]. For 2-year-old children, their preferences for fairness were similar when the alternative options [(2, 0) and (1, 0)] were different. However, there was a trend in 3-year-old children to be more likely to consider the other’s benefit when their own payoff was the same [*t*(15) = -1.732, *p* = 0.10, *d* = 1.1]. The small sample might have contributed to the absence of significance in 3-year-old children in terms of the value of index *d*. In the other-advantageous conditions, children always preferred fairness [*F*(1,34) = 3.62, *p* > 0.05, $\eta_{p}^{2}$ = 0.10].

### Discussion

This study aimed to examine the development of fairness preferences in 2- and 3-year-old Chinese children as distributors and recipients. We hypothesized that young children would allocate resources equally when they were distributors, that the preference for fairness would increase with age, and that children as young as 2 years old would prefer proposers who equally allocated resources when they were the recipient. It was found that children displayed fairness preferences early, as young as 2 years old. Young children’s fairness preferences increased with age and were influenced by distribution contexts.

#### Fairness Preference When Children Were the Distributors

Young children’s preference for fairness increased with age both when distributing resources between self and friend and distributing between two friends. Moreover, 3-year-old children displayed higher levels of fair behavior than the level of chance in both conditions, whereas 2-year-old children performed randomly in conditions of distributing resources between themselves and their friend but significantly preferred to distribute equally in conditions of distributing resources between two friends. The results suggested an early onset of inequity aversion (Blake and McAuliffe, 2011; Paulus et al., 2013) and provided evidence of inequity aversion based on young children’s actual fair behavior, not only on fair knowledge (Sloane et al., 2012; Sommerville et al., 2013). Moreover, an increasing preference for fairness over the course of early childhood was also evident, similar to the findings in Western cultures (Eisenberg et al., 1998; Fehr et al., 2008a; Shaw et al., 2014). These findings suggest that there is cross-cultural consistency in the early onset of inequity aversion and increasing fairness preference in young children.

In addition, young children were more likely to prefer fairness when they distributed resources between two friends than when they distributed resources between themselves and their friend. This might be due to the fact that self-interest served as an important motivating factor when they distributed resources between themselves and a friend. The difficulties in inhibiting their strong desire for candies might have led them to show a lower level of fairness preference (Blake et al., 2014). On the other hand, preference for fairness has typically been measured by children’s judgments regarding how to allocate resources between third parties (Damon, 1979), and thus the self-interest motive is excluded by this method. Our study examined young children’s preference for fairness as a third party as well, and we found that young children displayed a high level of fairness preference when they distributed resources between two friends, especially in 3-year-old children. The attempts of young children to achieve equal distributions when they were third parties provided evidence for a strong inequity aversion (Paulus, 2015).

#### Fairness Preference when Children were the Recipients

We found that young children’s preferences for fairness differed in the four distribution scenarios when they were the recipient; specifically, (0,2) as the alternative option was more likely to motivate young children to select a fair choice. Moreover, there was a trend toward young children’s preference for fairness in the other-advantageous condition than in the self-advantageous condition. Children were also more likely to prefer fairness with fewer payoffs in the other-advantageous condition. In other words, children tended to be more likely to show inequity aversion when they were in a disadvantageous position than when they were in an advantageous position. This result is similar to the findings of previous studies. For example, Birch and Billman (1986) found that 3-year-old children might notice and be averse to disadvantageous inequality in distributions. In addition, children 4–7 years old accepted allocations that put themselves at a relatively advantageous position and rejected those that put themselves at a relatively disadvantageous position (Blake and McAuliffe, 2011; Sheskin et al., 2014).

Although no significant differences in the preference for fairness were found between 2- and 3-year-old children when they were the recipients, further analysis showed that 3-year-old children were more likely to consider others’ benefit when their own payoff was the same in the self-advantageous condition. In the condition (1,1)-(1,0), called the “prosocial game” in Fehr et al.’s (2008a) study, children’s payoff was the same, while others’ payoff was different. Three-year-old children were more likely to consider others’ interest and show other-regarding preferences than 2-year-old children. Children were at an advantageous position in the (1,1)-(2,0) conditions, and they had to inhibit their own strong desire for candy and thus incur costs to behave fairly. In total, 58% of the 2-year-olds and 47% of the 3-year-old children preferred fairness in this condition, which both did not differ from chance (50%). Thus, we argued that children as young as 2 years old might already have developed a fairness preference and not be completely self-interested. In the meantime, this conclusion should be considered cautiously, and additional research should be conducted to further test the robustness of the finding.

For 2-year-old children, they were more likely to prefer fairness in the (1,1)-(1,2) condition, called the “envy game” in Fehr et al.’s (2008a) study, than in the (1,1)-(1,0) condition. Children’s payoffs remained the same, but the other’s payoffs were greater than their own in the (1,1)-(1,2) condition and less than their own in the (1,1)-(1,0) condition. Two-year-old children may already know how to avoid disadvantages in fairness by comparing their own payoff to others’ and may make decisions based on their relative advantage rather than focusing solely on their own gains (Blake et al., 2014). The concern for a relative advantage may prevent children from acting on their fairness knowledge when they actually allocate resources, especially in younger children. As the ERC (Equity, Reciprocity, and Competition) model states, competition in social interactions is highly considered by individuals (Bolton and Ockenfels, 2000). It is argued that competition may render fairness considerations irrelevant when there is no opportunity to punish the monopolist (Fehr and Schmidt, 1999).

Children younger than 2 years old already display a fairness preference (Sloane et al., 2012; Sommerville et al., 2013). In the present study, although 2-year-old children began to prefer fairness in some conditions, their performance in some conditions, such as distributing resources between self and friend as a distributor and distributing resources in self-advantageous conditions as recipients, was similar to that of the random level. The difference might be due to the fact that knowledge of fairness was examined in Sloane et al.’s (2012) and Sommerville et al.’s (2013) study, whereas fair behavior through participant involvement was examined in our study. This gap between fairness knowledge and fair behavior might be due to children’s desire to maintain an advantageous position compared to their peers (Blake et al., 2014). Moreover, this gap is argued to be motivated in children by a context of windfall gains, in which strategic concerns with maintaining an advantageous position relative to their peers appear to prevent children from following fairness norms (Blake et al., 2014). As in our study, the resources were given by the experimenter and were not earned by the participants. It is predicted that children younger than 2 years old might be able to apply fairness norms in the context of gaining resources through collaborative efforts. In addition, a weak executive function may prevent young children from inhibiting their desires for the resources (i.e., candy) when they are in charge of the distribution, and they are accordingly unable to follow the fairness norms that they already know (Blake et al., 2014).

#### Limitations and Implications

Fewer 3-year-olds than 2-year-olds participated in this study. A larger sample size of participants should be recruited, time and resources permitting, to increase the statistical power of testing the developmental differences in fairness preferences of 2- and 3-year-old children.

We would have found it interesting to examine the development of fairness preferences in children. However, only cross-sectional data were collected in this study. Longitudinal designs and analysis will be a future direction of this research.

Young Chinese children’s fairness preferences were examined in the study. This is one of the few studies conducted in a non-Western sample. Future studies should increase the scientific knowledge regarding child development in different cultural contexts.

Children as young as 2 years old already displayed fairness preferences in our study. To provide evidence for the ontogenetic origins of fairness, future research can examine whether the preference for fairness emerges in even younger children. Eye tracking technology is an effective way to investigate psychological mechanisms in young children, even in non-verbal infants. Future studies should adopt eye tracking technology to investigate the evolutionary origins of fairness preferences in younger children.

## Conclusion

When young children allocated resources as the distributor, their preference for fairness increased with age, and they were more likely to prefer fairness when they distributed resources between two friends than when they distributed resources between themselves and a friend. Our results suggest that even young children (2-year-olds) display inequity aversion and this preference for fairness increases over the course of early childhood.

When young children were distributed resources as the recipients, their preference for fairness differed in the four distribution scenarios. There was a trend in young children’s preferences for fairness in the other-advantageous condition in comparison with the self-advantageous condition. That is, children may tend to be more likely to display inequity aversion when they are in a disadvantageous position.

## Author Contributions

WW, JL, and LZ designed the experiment. WW collected the data. JL analyzed the data. JL, WW, JY, and LZ wrote the manuscript.

## Conflict of Interest Statement

The authors declare that the research was conducted in the absence of any commercial or financial relationships that could be construed as a potential conflict of interest.
